# Clinical amelioration of severe low back pain in lumbar spondylosis using selective siddha therapeutics - A case report

**DOI:** 10.6026/973206300222920

**Published:** 2026-05-31

**Authors:** Selvaraj Jothilingam, Krishnaaveni Damodaran, Shakthi Paargavi Ambalavanan, Gayatri R, Srinivasan Venkatachalam, Ramamurthy Murugan, Elansekaran Selladurai, Christian Gnanaraj Johnson

**Affiliations:** 1 Department of Noi Naadal, National Institute of Siddha, Tambaram Sanatorium, Tamil Nadu, India

**Keywords:** Siddha, Lumbar spondylosis, Peechu, Puravalayam, Navarakizhi ottradam, Thandagavatham

## Abstract

Thandagavatham is one of the 80 types of Vatha diseases mentioned in Siddha literature and it is comparable to Lumbar spondylosis, a degenerative disorder of the lumbar spine in western medicine. The conventional treatment often fails in providing long term relief from pain despite the high cost of treatment. Therefore, it is of interest to report the successful treatment of Thandagavatham using a combined selected Siddha Internal and external therapy regimen providing a long-lasting relief in a shorter duration. A 42-year-old male presented to Ayothidoss Pandithar Hospital with severe lower back pain for 7 months and was diagnosed with Thandagavatham through Siddha and modern diagnostic parameters. The patient was administered 26 days of Siddha inpatient treatments including Inpatient Department (IPD) medicines, Therapeutic enemata, Oil Damming, Medicated grain fomentation and Poultice therapies followed by 38 days of internal medications in Out Patient Department (OPD). The Modified Oswestry Low Back Pain Index decreased from 70% at baseline to 7% after treatment and the Visual Analog Scale score reduced from 5/10 to 1/10 by the end of the treatment period. The patient remained symptom free even after an 8-month drugless follow-up period, suggesting that Siddha external therapies combined with internal medicines may shorten the treatment duration and provide long-term relief.

## Background:

Lumbar Spondylosis (LS) is a degenerative condition affecting the discs, vertebral bodies and associated joints of the lumbar spine leading to low back pain. Low back pain is the most common musculoskeletal problem encountered globally [1]. Low back pain affects approximately 60 - 85 % of adults during some point in their lives [2, 3, 4]. About 619 million people were affected globally by low back pain in the year 2020, estimated from the systematic analysis of global Burden of disease study [5]. Treatment with NSAIDs, pain management medications, surgical interventions such as spinal decompression, nerve root decompression and spinal fusion etc., are not giving promising results [6, 7, 8]. So, there is a rise in global trend towards the use of complementary and alternative medicine. Siddha system of medicine is one of the traditional systems of India, which has its roots from ancient Tamil civilization [9]. Sage *Agathiyar* is considered as Father of Siddha medicine and has classified diseases of humanity into 4448 types in his treatise *Agathiyar Rathina Churukka Naadi Nool*. The diseases associated with pain and disabilities are usually classified into 80 types of *Vatha* disease. One of these types is *Thandagavatham,* which affects the Lumbar spine caused primarily due to vitiation of Vatham Humour [10]. The disease manifestation and pathology of Lumbar spondylosis (ICD 11 CODE: SP42) can be correlated to the term *"Thandagavatham"* mentioned in classical Siddha literature (NAMC CODE: Z34 & ISMT: 4.24.76) [11, 12, 13]. In Siddha system of medicine, vitiated *vatham* humour is usually pacified by giving purgation therapy (Viresanam) which is evident from the quote *"Viresanathaal vatham thaazhum"*. It has been hypothesized that the Purgation exerts its action by regulating *Abaana vaayu* (Downward force), which is predominantly acting in large intestine part of the gut [14]. According to *Theraiyar Vagadam*, it is also emphasized that the Vatham diseases that are not responding to internal medications can be treated by Therapeutic enemata *(Peechu)* with external oils which directly act on the large intestine part of the gut where *Abaana vaayu* resides to pacify *vatham* [15]. Therapeutic enemata, Oil damming *(Puravalaiyam)*, Medicated grain fomentation *(Navarakizhi ottradam), Aavarai ulunthu* poultice are commonly used external medicines documented in Siddha practice at National Institute of Siddha. Oil Damming which is also called *Ennai Uural* is used to pacify the *Vatham* in affected area. Medicated grain fomentation which is also known as *Thaniya pasai* is indicated for treating pain in joints [16]. Modified Oswestry low back pain Index (ODI) was used to assess the prognosis before and after the treatment [17, 18]. Therefore, it is of interest to report the successful treatment of Thandagavatham with a combined regimen of regular Internal medicines of NIS IPD along with these external therapies using Modified Oswestry low back pain Index (ODI).

## Patient information:

A 42-year-old man, who is working as a school teacher had visited OPD at NIS with complaints of a severe pain in low back and calf muscle region of left limb on 14.08.2023. The pain prevented him from sitting and standing for more than 5 mins and had difficulty falling asleep due to lower back pain. Also, the patient had complaints of pain in cervical region with restricted range of motion and the pain used to get aggravated on flexion and extension of neck. He was a non-smoker, non-alcoholic and had no relevant family history. Patient had a history of fall over staircase landing in sitting position before 3 years, after which the symptoms developed gradually and experienced a severe pain last 7 months. Initially he took allopathic pain-relieving medication from the nearby clinic for 20 days but reported no improvement in the condition. The patient was admitted to Inpatient Department (IPD) of Ayothidoss Pandithar hospital, National Institute of Siddha on 14.08.2023 the prognosis of which is reported here.

## Clinical findings:

The patient was clinically examined and found to have a mild swelling in the sacroiliac region. Had a decreased range of movements in the cervical region. Positive Straight leg raising test was positive with pain felt at 40o flexion in left lower limb. The baseline Modified Oswestry Low Back Pain Index was 70%. The findings of the clinical examination of the patient are shown in Table 1. Assessment based on Siddha principles showed deranged *Vatham (Viyaanan, Samaanan), Pitham (Saathagam) and Kabam (Avalambagam, Kilethagam)*. Wrist circumetric sign *(Manikadainool)* measurement was found to be 7 finger breadths at baseline. *Vatha Pitham Naadi* was recorded at admissions which are depicted in Table 2.

## Diagnostic assessment:

**Figure 1:**
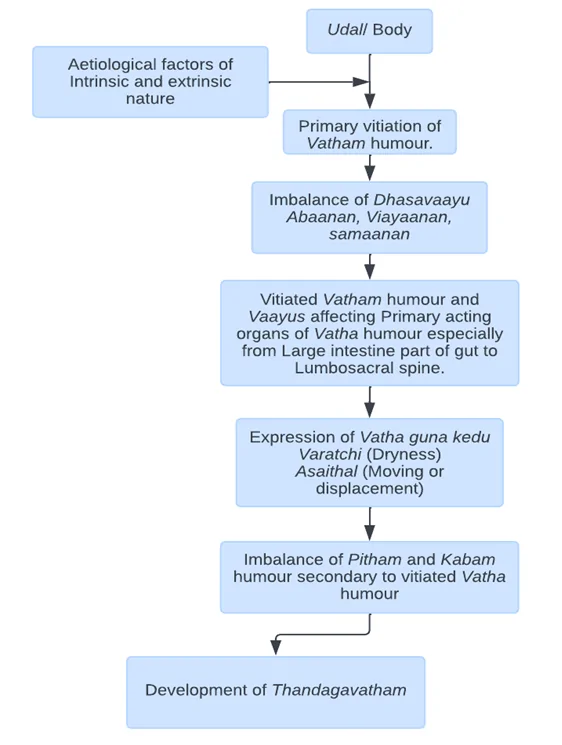
Pathogenesis of Thandagavatham

Magnetic resonance imaging of the whole spine showed a loss in normal cervical lordosis and chronic degenerative changes in all the cervical, dorsal and lumbar intervertebral discs. Other finding was chronic diffuse posterior disc bulge with posterior and bilateral vertebral osteophytes causing indentation on the spinal canal and central thecal sac at the level of C4 - C5, C5 - C6, C6 - C7 levels. Posterior disc bulges causing indentation on the spinal canal and bilateral neural foraminae of L4 - L5, L5 - S1; Bilateral facetal joint hypertrophy with ligamentum flavum thickening; Anterior spondylotic changes at all the cervical, dorsal and lumbar levels; Dorsal angulation of distal coccygeal segments and bilateral sacroiliitis on posterio inferior aspects. Based on the symptoms and clinical findings the patient was diagnosed with Low back pain due to Lumbar Spondylosis - Thandagavatham. Pathogenesis: *Thandagavatham* is one of the *Vatha* diseases. It is primarily caused due to the vitiation of *Vatha* humour by various aetiological factors that can vitiate *Vatha* humour. The brief pathogenesis of *Thandagavatham* according to Siddha literature is depicted in the Figure 1.

## Timeline:

**Figure 2:**
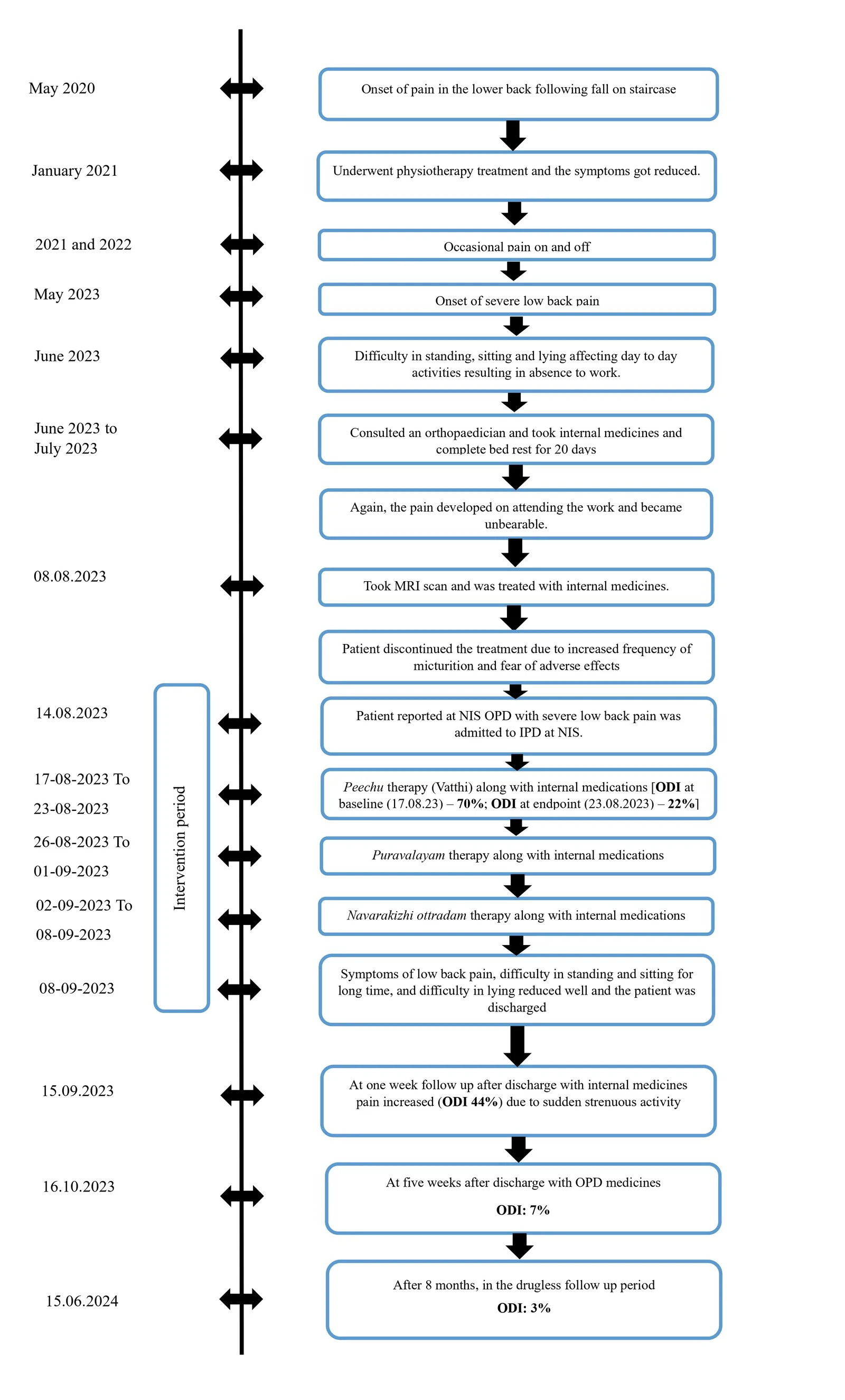
Timeline

The timeline of events from development of disease to completion of 8 months of drugless follow-up was depicted in Figure 2.

## Therapeutic interventions:

The patient was first administered Therapeutic enema with *Chitramutti thailam* [19], 50 ml for 7 consecutive days along with Internal Siddha medicines, daily external application of medicated pain oil and Poultice application with *Cassia auriculata* leaves and *Vigna mungo* (Black gram) powder. The whole therapeutic regimens during the 26 days of IPD admission are tabulated in Table 3.

## Therapeutic enemata:

Therapeutic enemata was done based on the standard operating procedure. The patient was prepared for the therapy at morning time immediately after having breakfast. Gentle massage with *Chitramutti thailam* over abdomen and back of the trunk was done for the relaxation of the patient. Fomentation with hot water was then done over patient's abdomen and para spinal area. 50 ml of *Chitramutti thailam* was then heated in hot water bath to a lukewarm state and drawn into an enema syringe. The thailam was pushed slowly as enemata. Patient was then advised to lie still for few minutes and given a gentle massage over the abdomen and back. The procedure was repeated for 7 days.

## Oil damming:

Black gram powder was mixed with sufficient quantity of water to make a thick paste. It was rolled into a ring. The patient was made to lie on a prone position exposing the lower back region. Then, using the thick dough ring, a rim was made and fixed firmly on the low back (lumbo-sacral) region. *Laghuvidamutti thailam* was heated passively with double boiling method and poured little by little within the rims of the dough bund to avoid the unexpected discomfort due to heated oil. Care was being taken to prevent the leakage of oil. When oil cools down, the oil was mopped out using cotton, warmed up again and refilled. Temperature of oil was maintained uniformly throughout the procedure by replacing it with warm oil [16].

## Medicated grain fomentation:

*Navara* variety of rice was boiled along with roots of *Sida acuta Linn* with sufficient quantity of milk and equal amount of water till the rice was fully cooked. The boiled rice was then mashed with the fruit of *Musa paradisiaca Linn* and made into a fomentation bundle *(Kizhi)*. The fomentation was done in a circular motion all over the body by dipping the fomentation bundle in milk drained from the cooked rice in a Luke warm consistency.

## Poultice:

*Cassia auriculata* leaves powder with double the quantity of *Vigna mungo* seed powder was made into a paste by using egg white and was then applied on the lumbosacral region as a poultice for 3 hours every day.

## Outcome and follow-up:

The patient was monitored closely day by day for the improvement of symptoms. The patient showed good prognosis of symptoms after few days of starting Therapeutic enemata. The patient who could sit barely for 5 minutes was able to sit in seat for more than 30 minutes. However, the discomfort in lying position persisted. But at the end of 7 days, the pain during lying position also reduced well. The severity of the disability based on the ODI scale before the starting of the treatment was found to be 70%. At the end of 7 days treatment, patient showed good improvement of the symptoms and had a score of 20% in ODI scale. The patient was then treated with regular IPD medicines, Oil damming and *Aavarai ulunthu* poultice, followed by Medicated grain fomentation procedure over 16 days. The patient was then discharged on 08-09-2023 and advised to follow up in OPD. The patient then visited OPD on 15-09-2023 for follow up. He was assessed by using ODI scale, had a score of 44%. The patient continued taking internal medicines in OPD for a month. After which the ODI score further reduced to 7%. The patient then discontinued the treatment and after 8 months of drugless follow-up period, he felt only a minimal discomfort only when lifting heavy weights and the ODI score reduced to 3%. The Prognosis of ODI Score and Pain Scale over Time in Relation to Siddha intervention and Follow-Up are tabulated (Table 4). The patient adhered to the treatment and no adverse events occurred during the treatment.

## Discussion:

Thandagavatham is caused due to the vitiation of Vatham humour which predominantly presents in lumbosacral spine, stools, nerves and Abaanan etc. [21]. The movement of deranged *Abaana Vaayu* from *Moolathaaram* up to the point of lumbar spine leads to degeneration. The Vatham humour is associated with the large intestine part of the gut where the Abaana Vaayu exerts its action. So, the therapy is targeted towards the large intestine. The vitiated Vatham which is expressed as dryness *(Varatchi)* of the spinal intervertebral discs, bulge, protrusion or prolapse of the discs (Asaithal) can be ameliorated by treatment with medicines having unctuous *(Neippu)* property through Oleation, external therapies through drugs with hot potency *(Veppam)* and cold potency *(Seedha veeriyam)* having Stabilizing *(Sthiram)* properties. *Chitramutti thailam* indicated for Vatham diseases has been administered as Therapeutic enemata for this patient. The key ingredients of *Chitramutti thailam, Sida cordifolia Linn* and sesame oil both have potent analgesic and anti-inflammatory activities [22, 23]. *Sida cordifolia Linn* has a sweet taste, Cold potency and emollient properties [24]. *Chitramutti thailam* has an overall cold potency and unctuous property (Oleation). It helps in reducing the Unstable *(Asaithal)*, light *(Ilagu)*, dry nature of aggravated Vatham. Oil damming with Laghuvidamutti thailam is used for its unctuous property and hot potency [10]. The key ingredient of *Laghuvidamutti thailam, Strychnous nux-vomica Linn* has nervine tonic property [25]. Medicated grain fomentation has a good nourishing property and was indicated for various types of joint pain [16, 26]. Stability of the intervertebral discs can be established by *Aavarai ulunthu* poultice [27]. These external therapies are cost effective, non-invasive and did not require much man power. It also reduced the load of internal medicines and better results were obtained within few days of starting the treatment. The patient was suffering from moderate low back pain for three years of which it was severe so much so affecting the day to day activities for 7 months before presenting to Siddha treatment. During the severe presentation of pain, patient had been treated with internal biomedicines, (Bromelain + Trypsin + Rutoside Tab (Phlogam), Alpha Lipoic Acid-100mg + Folic Acid-1.5mg + Mecobalamin-1500mcg + Vitamin B6-3mg + Vitamin D3-1000iu Tab (Nervesmile), Pregabalin 25mg (A2D) capsule, Amitriptyline 10mg (Tryptomer) Tab, Alpha Lipoic Acid + Superoxide Dismutase (Alsody) Tab) and bed rest for 20 days from a nearby Orthopaedic consultant and had his symptoms relieved to some extent. However, the pain became severe on resuming the work. Then he reported at NIS OPD during the month of August 2023. After treatment with Siddha Internal medicines and External therapies, there was a good reduction of Low back pain in a period of 26 days of IPD treatment which is evident by reduction in visual analogue scale from 5/10 at baseline during admission to 2/10 during discharge. Post admission, he was treated in continuum for 45 days and the pain subsided fully with only occasional mild pain on lifting heavy objects. The post treatment follow-up revealed that the pain did not worsen even after 8 months of discontinuing treatment. The effectiveness of the treatment was evident from initial period that the ODI score came down from 70 % to 22% after 7 days of Therapeutic enemata. Major complaints such as severe low back pain which was aggravated on sitting and standing even for 5 minutes gradually improved and on the due course after 2 weeks, the patient was able to sit and stand for more than 30 minutes without any discomfort. But the discomfort in lying position persisted over a period of time, which was also reported to be relieved in further follow up visits. On resuming to work there was a transient increase in ODI score to 44 %. This might be ascribed to abrupt exertion after a prolonged period of bed rest. Further down the period, with internal medicines in the NIS OPD, the pain and discomfort reduced much (ODI score: 7%). After 8 months of drugless follow up the pain was reported to be relieved well (ODI score 3%). In a similar case report, Shenbagaraj *et al.* documented significant clinical improvement in Thandagavatham following 48 days of Siddha Varmam therapy, with a marked reduction in pain intensity (VAS 8→3) and functional disability (Oswestry Disability Index 66%→18%), and importantly, the benefits were sustained without symptom recurrence during a six-month follow-up period, supporting the stabilizing effect of Siddha external therapies on vitiated Vatham [28]. Vinubharathi *et al.* studied the effect of standalone *Aavarai ulunthu* pattru in the management of Thandagavatham in 35 patients, reported a notable reduction in pain severity. Overall, 14 % showed good improvement, 37 % moderate improvement, and 32 % mild improvement [27]. These articles support the observations of our case report on Thandagavatham, that the Siddha external therapies along with internal medicines may has a huge potential in management of Thandagavatham in a more cost-effective manner. This observation with *Chittramutti thailam* enema about its usefulness is reported so comprehensively for the first time and this reporting provides a lead for further study on a larger scale for estimating the efficacy of this treatment regimen.

## Patient perspective:

Patient felt a sense of relief from the pain after few days of starting the treatment and adhered to the treatment well as he had prompt relief. He admitted on further follow up that; he was able do more stressful jobs with much less discomfort than before.

## Informed consent:

Informed consent was obtained from the patient to publish the report.

## Abbreviations:

B.I.D: Twice a day

DALY: Disability-adjusted life years

G: Grams

IPD: In-patient Department

LBP: Low Back Pain

ML: Millilitre

MRI Scan: Magnetic Resonance Imaging Scan

NIS: National Institute of Siddha

NSAIDs: Non-steroidal anti-inflammatory drugs

ODI: Oswestry Disability Index

OPD: Outpatient Department

T.I.D: Thrice a day

TV: Thandagavatham

WHO: World Health Organisation

## Figures and Tables

**Table 1:** Therapeutic intervention during the stay in IPD

**Clinical examination**	**Findings**
Inspection	Whole spine:
	Symmetrical in nature
	No scar
	No deformity,
	No atrophy
Palpation	Normal texture and thickness of skin
	Tenderness present
Straight leg raising test	40 ° in Left Lower Limb
FNST	Negative
Scober test	Negative
Lassegue's test	Negative
Drawers test	Negative
Lhermitte's sign	Negative
Spurling test	Positive
Shoulder abduction test	Negative
Chin Test	Positive
Faber test	Negative

**Table 2:** Evaluation based on Siddha Eight - fold diagnostic principles

**Siddha Clinical Examination**	**Findings**
Pulse	Pithavatham
Tactile Examination	Normal (Mithaveppam)
Tongue	Normal
Colour	Mixed type (Thontha niram)
Voice/ speech	Normal speech (Sama oli)
Eye examination	Normal
Stool examination	Regular
**Urine:**	
Colour of the urine	Pale yellow colour
Oil drop on urine sign	Round shape
Wrist circummetric sign	7
Panchapatchi Calculation	Waning phase of Moon
(Five birds Sign)	(Theypirai Thingal) 14/08/23 10:00 am

**Table 3:** Therapeutic intervention during the stay in IPD

**Date**	**Treatment**	**Dose**
14-08-2023	Admission	
14-08-2023	Sagala Noi chooranam [20]	2 g b.i.d, with Hot water (after food)
	Sangu parpam Tablet	2 b.i.d, with Milk (after food)
	Arumuga chendooram	200 mg b.i.d, with Honey (after food)
	Vathakesari thailam	External application
	Karpoorathy thailam	External application
15-08-2023	Elathy chooranam Tablet	2 t.i.d, with Hot water (after food)
	Sangu parpam Tablet	2 b.i.d, with Milk (after food)
	Thurunji manapaagu	5 ml b.i.d, with Hot water (after food)
	Karpoorathy thylam	External application
16-08-2023	Elathy chooranam Tablet	2 t.i.d, with Hot water (before food)
To	Amukkura chooranam Tablet	2 t.i.d, with Hot water (after food)
1/9/2023	Muthuchippi parpam Tablet	2 t.i.d, with Hot water (after food)
	Karuppu vishnu chakram Tablet	2 b.i.d, with Honey (after food)
	Laghuvidamutti thailam	External application
	Pain balm	External application
17-08-2023 To 23-08-2023	Chitramutti thailam - Therapeutic enemata	50 ml.
25-08-2023 To 31-08-2023	Oil damming with Laghuvidamutti thailam	Sufficient quantity
2/9/2023 To 8/9/2023	Medicated grain fomentation	Sufficient quantity
16-08-2023 To 8/9/2023	Aavarai ulunthu Poultice	Sufficient quantity
8/9/2023	Patient's condition was improved and discharged. Advised to follow up in OPD	

**Table 4:** Progress of ODI score and pain scale over time with siddha intervention and follow up.

**Date**		**ODI Score**	**Pain scale**
14.08.2023	On admission		10-May
17.08.2023	Before administering Therapeutic enemata (Peechu therapy)	70%	
23.08.2023	After seven days of Peechu therapy along with internal medicines.	22%	
08.09.2023	On discharge		10-Feb
15.09.2023	On one week follow up after discharge.	44%	
16.10.2023	After 38 days of discharge with OPD medicines	7%	10-Jan
15.06.2024	After 8 months of drugless follow up period	3%	10-Jan
